# Reducing the harmful effects of noise on the human environment. Sound insulation of industrial skeleton enclosures in the 10–40 kHz frequency range

**DOI:** 10.1007/s40201-020-00560-2

**Published:** 2020-10-15

**Authors:** Witold Mikulski

**Affiliations:** Central Institute For Labour Protection — National Research Institute, 16 Czerniakowska Str., Warsaw, Poland

**Keywords:** Sound insulation, Acoustic enclosures, Soundproof enclosure, Ultrasound, Sound power level

## Abstract

**Purpose:**

The purpose of the research is to work out a method for determining the sound insulation of acoustic enclosures for industrial sources emitting noise in the frequency range of 10–40 kHz and apply the method to measure the sound insulation of acoustic enclosures build of different materials.

**Methods:**

The method is developed by appropriate adaptation of techniques applicable currently for sound frequencies of up to 10 kHz. The sound insulation of example enclosures is determined with the use of this newly developed method.

**Results:**

The research results indicate that enclosures (made of polycarbonate, plexiglass, sheet aluminium, sheet steel, plywood, and composite materials) enable reducing the sound pressure level in the environment for the frequency of 10 kHz by 19–25 dB with the reduction increasing to 40–48 dB for the frequency of 40 Hz. The sound insulation of acoustic enclosures with a sound-absorbing material inside reaches about 38 dB for the frequency of 10 kHz and about 63 dB for the frequency of 40 kHz.

**Conclusion:**

Some pieces of equipment installed in the work environment are sources of noise emitted in the 10–40 kHz frequency range with the intensity which can be high enough to be harmful to humans. The most effective technical reduction of the associated risks are acoustic enclosures for such noise sources. The sound pressure level reduction obtained after provision of an enclosure depends on its design (shape, size, material, and thickness of walls) and the noise source frequency spectrum. Realistically available noise reduction values may exceed 60 dB.

## Introduction

In their everyday life and work environment, humans are exposed to a plurality of harmful and uncomfortable factors. The noise is defined as sounds (acoustic vibrations) showing undesirable and/or harmful effect on humans or making it difficult to carry out specific activities. Such sounds reach humans as airborne stimuli. The fundamental parameter characterising quantitatively the intensity of sonic phenomena (including noise) in the frequency range 20–20,000 Hz is the A-weighted sound pressure level. Any noise characterised with values of that parameter exceeding 80 dB [1–7] can be the cause of permanent damage of hearing in a human being. In Poland, the A-weighted sound pressure level of 85 dB is adopted as the so-called maximum admissible intensity (MAI) applicable in the context of the hearing protection [2, 3]. Any noise with the A-weighted sound pressure level falling in the range 55–75 dB can be detrimental to or prevent the exposed person from carrying out certain activities, including work-related ones (such as verbal communication) [4, 5]. In Poland, admissible values of the A-weighted sound pressure level in the context of the employee’s possibility to fulfilling their basic tasks depend on the nature of the work and are included in the range 55–75 dB [3]). In the everyday life environment, any noise with the A-weighted sound pressure level in the range 25–40 dB can make it difficult to have a rest, prevent from sleeping, etc. [4]. When the parameter used to determine intensity of sound is the A-weighted sound pressure level (and the quantities derived from it such as, for instance, the equivalent A-weighted sound pressure level or the A-weighted noise exposure level), the so-called audible sounds are being taken into account with frequencies falling into the range 20–20,000 Hz [3–5]. That limitation is adopted universally as it encompasses the vast majority of cases involving harmful effects of sounds on humans. However, such approach lacks completeness which could be reached only if all cases of undesirable or harmful effect of sound on humans are taken into account. Sounds with frequencies below 20 Hz (infrasounds) and sounds with frequencies higher than 20 kHz (ultrasounds), though inaudible to the human ear, can be still unpleasant, bothersome, or even harmful [2–8]. In the human out-of-work environment, sound pressure levels of infrasounds and ultrasounds are small enough to be taken into account only in relatively rare cases, especially in the context of their harmful effects on humans [2–8]. However, ultrasounds present in the human work environment can be characterised with values of the sound pressure level (the parameter determining intensity of sound in this range of frequencies) so high that their effect on workers can be harmful [3–8] and must not be ignored. In Poland, when harmful and uncomfortable factors in the human work environment depending on frequency of involved sound vibrations are considered, the noise is categorised into three ranges, of which the first is called the infrasonic noise (the frequency range 1–20 Hz), the second is referred to as the audible noise (the frequency range 20–20,000 Hz), and the third is named the ultrasonic noise (the frequency range of about 10,000–40,000 Hz) [2, 3, 6–10]. In Poland, the latter noise category encompasses all 1/3 octave frequency bands with mid-band frequencies falling into the range of 10–40 kHz [2, 3, 6–14]. In other countries, the 10–20 kHz range is called the very high frequency range whereas the bracket of higher frequencies is referred to as the ultrasonic range [3–5].

The present paper focuses on the issue of occupational exposure of workers to the ultrasonic noise. In Poland, allowable values concerning this type of noise (admissible equivalent sound pressure level in 1/3 octave band MAI) specified for workstations are 80 dB (in 1/3 octave band with centre frequencies 10, 12.5 and 16 kHz), 90 dB (20 kHz), 105 dB (25 kHz), and 110 dB (31.5 and 40 kHz) [2, 3]. Admissible maximum sound pressure level MAI in Poland are by 20 dB larger than the equivalent value [2, 3]. Lower MAI values apply to pregnant women and juveniles. In other countries, these values, although not identical, are similar [3, 4]. Evaluation of noise affecting humans in the work environment consists in determining whether the noise occurring at a specific workstation (measured values of parameters characterising noise) exceeds the levels quoted above (is acceptable/admissible) or not (is unacceptable/inadmissible).

The ultrasounds at workstations come from process ultrasonic sources (which generate ultrasounds intentionally to realise a technological process, e.g. heating an article) and from other devices in which the emission of ultrasounds is not an intended effect of the actually realised process [4, 6–9]. The process sources of the first type are the cause of significantly more intense ultrasonic noise compared to the other and are used in various technological processes, such as cleaning of materials (including mainly washing in liquids but also mechanical cleaning in, for instance, dental scale removers), erosion drilling, disintegration of materials (including production of powders in atomizers), welding of metals and non-metals, sterilisation (in medicine and food industry), blanking and piercing (including patterns in plastic films), cutting (in e.g. food industry), soldering, etc. [4, 6, 7, 9, 10]. The ultrasonic devices which use ultrasonic energy in the production process include: ultrasonic washers, ultrasonic welders (for plastic, metal and hardly wieldable materials), manual soldering units, ultrasonic crucibles, fabric treatment machines (jet machines, lace machines, and quilting machines), dental devices used for tartar removing (called scalers), ultrasonic guillotines, ultrasonic knives or ultrasonic curtains, ultrasonic erosion machines, and ultrasonic drills [4, 6, 7, 10]. The ultrasonic noise emitted by the devices has, in the majority of cases, a narrow-band spectrum corresponding to the frequency range in which the installed exciter of ultrasonic vibrations is operated [6–10]. For instance, exciters used in ultrasonic welders work typically with frequencies of 20, 25, 36, and 40 kHz. In many cases, ultrasonic noise level indicators (the equivalent sound pressure level in 1/3 octave band and/or the admissible maximum sound pressure level) at workstations of operators of the devices exceed the above-quoted maximum admissible intensity levels MAI (in Poland, [2, 3]). Some of these devices can be operated in sealed (fully closed) sound-insulating enclosures [11] and then ultrasonic noise outside such enclosure is, in the majority of cases, very small and negligible from the point of view of its harmful effect on humans [4, 5]. Therefore, fully sealed enclosures (especially those provided with an inner lining of sound absorbing material) turn out to be a very effective measure of ultrasonic sound reduction [11–22]. The parameter characterising acoustic effectiveness of an enclosure in scope of its ability to attenuate the sound penetrating through it is called “the sound insulation of acoustic enclosure” (or “the sound insulation of enclosure”) in applicable standards and literature of the subject [11, 15–21]. The value of the sound insulation of a given enclosure depends not only on its structure and materials of which it is constructed but also on the frequency spectrum of sound emitted by the enclosed noise source [11, 18–21]. Therefore, to design an effective sound-insulating enclosure for a specific device it is necessary to know sound insulation properties of enclosure walls and parameters characterising the noise emission from the source. Unfortunately, acoustic properties of sound-insulating enclosures (sound insulation of enclosure walls, but also the coefficient of absorption of materials used for enclosure inner linings) remains still a non-standardised quantity in the frequency range above 10 kHz [11–28]. There is also no common agreement on methods for determining the parameters in this frequency range. Moreover, to date there exists no commonly accepted standard for evaluation of the parameter fundamental for determining the sound insulation of enclosure which is the sound power level of a source in the frequency range below 20 kHz ([9, 10, 22–26]). That is a significant gap in the knowledge making impossible to carry out effective activities aimed at reduction of harmful effects of ultrasonic noise on humans. However, as measurements concerning such noise at workstations reveal that the admissible levels are exceeded, some manufacturers of ultrasonic equipment, despite the above-mentioned incompleteness of the knowledge, strive to design, construct, and apply enclosures for their products on an ad hoc basis through trial and error. The effectiveness of these solutions is verified by taking measurements of noise at workstations. Although casual and accidental, such methods turn out to be effective in some cases. On the other hand, it must not be assumed that the acoustic effectiveness of a soundproof enclosure demonstrated for a specific noise source will be the same for another device, as different devices may (and in the majority of cases actually do) emit the ultrasonic noise of different frequencies which has an effect on effectiveness of attenuation of sound penetrating through the enclosures. It follows from the above that there is the need to initiate extensive research projects oriented at working out methods for determining, on one hand, emission of noise from devices, and on the other, properties of materials and enclosures (in the aspect of both sound-absorbing and sound-insulating properties) in the frequency range of 10–40 kHz. Research projects of that type have been already undertaken in such areas as the noise emissions from devices, sound absorbing properties of materials, and acoustic insulation performance of enclosures.

In case of enclosures it is also possible to adopt another approach. One may develop a method for determining the sound insulation of enclosure and other sound insulation properties for typical soundproof enclosures (with walls made of various materials with different thickness) in the whole frequency range of interest by using a standard broad-band sound source. Results of such tests would enable determining the effect of the type and thickness of materials used to construct the enclosures on their sound-insulating properties. Given the test data and gaining knowledge of the emission from an ultrasonic noise source or immission of ultrasonic noise at a workstation, it would be possible to select the most suitable sound-insulating enclosure. Such approach was adopted in the study reported in the present paper.

When determining the sound insulation of soundproof enclosures, the typical design solution was selected for examination, namely the skeleton structure with walls made of various materials such as sheet steel or aluminium, polycarbonate or plexiglass boards, or plywood. The methods for determining the sound power level of source without and with enclosure and the sound insulation of enclosure were developed with the specificity of sound radiation and propagation in the frequency range above 10 kHz taken into account (including, in particular, high directivity of sound radiation emitted by such sources and the significant effect of damping high-frequency sounds in air [9–12, 25, 29, 30]).

The article presents the method for determining the sound insulation of enclosures for a noise source emitting sound energy in the frequency range of 10–40 kHz (as a result of the author’s previous research [30]). The method is based on measurements of the insertion loss when the sound power level of a reference source is determined [9, 10, 24, 25, 30], the quantity being called “the sound power insulation” in [18, 19, 30] and determined in 1/3-octave frequency bands with centre frequencies of 10, 12.5, 16, 20, 25, 31.5, and 40 kHz.

Moreover, results are given of tests on the sound insulation of skeleton enclosures made of different materials with different thickness of acoustic enclosure walls and further, with and without some sound-absorbing material inside the enclosure [11–13, 30]. The objective of the tests was to specify the usefulness of various materials for walls of sound-absorbing and insulating skeleton enclosures for technological ultrasonic noise sources [14, 30].

The proposed methods for determining the sound power level for a noise source in the frequency range from 20 kHz to 40 kHz and the sound insulation of enclosures in the frequency range from 10 kHz to 40 kHz are novel. Results of testing skeleton enclosures walls of which are made of various materials with different thickness are also new as to date, acoustic properties of enclosures in the frequency range above 10 kHz were not determined (except for results concerning enclosures made of plywood which were published by the present author in [30]).

## Materials and methods

### Scope of research — Test samples

The subject-matter of the study was:

I. Comparing the sound insulation of skeleton enclosures with walls made of various materials (without sound-absorbing materials used inside the enclosure):

- single-layer polycarbonates PC, wall thickness of 5 mm (for short, abbreviated *PC5)*;
- single-layer poly(methyl methacrylate) PMMA, wall thickness of 5 mm (abbreviated *PMMA5*);
- single-layer sheet steel, wall thickness of 2 mm (abbreviated *STEEL2*);
- single-layer sheet aluminium, wall thickness of 2 mm (abbreviated *AL2*);
- single-layer plywood, wall thickness of 6 mm (abbreviated *PW6*);
- three-layer composite (sheet aluminium 0.3 mm + polyethylene 3.4 mm + sheet aluminium 0.3 mm) (abbreviated *ALPO4*).

II. Comparing the sound insulation of skeleton enclosures with walls made of aluminium and plywood of different thickness:

- single-layer sheet aluminium, wall thickness of 4 mm and 2 mm (abbreviated *AL4* and *AL2*);
- single-layer plywood, wall thickness of 12 mm and 6 mm (abbreviated *PW12* and *PW6*).

III. Comparing the sound insulation of skeleton enclosures with walls made of three materials, enclosures with and without inner lining of 15 mm polyurethane foam:

- single-layer sheet aluminium, wall thickness of 2 mm, without and with 15 mm foam lining (abbreviated *AL2* and *AL2 + FOAM*, respectively);
- single-layer plywood, wall thickness of 6 mm, without and with 15 mm foam lining (abbreviated *PW6* and *PW6 + FOAM*, respectively);
- three-layer composite (sheet aluminium 0.3 mm + polyethylene 3.4 mm + sheet aluminium 0.3 mm), without and with 15 mm foam lining (abbreviated *ALPO4* and *ALPO4 + FOAM*, respectively).

### The test method

The objective of the tests was to determine the effect of different materials used to construct acoustic enclosures and the impact of acoustic enclosure with lining of sound absorbing material on the sound reduction effectiveness of enclosures for noise sources emitting the sound in the frequency range of 10–40 kHz.

The test method involved comparing results of determination of the sound power insulation [11, 16–18, 21, 30] of a skeleton acoustic enclosure with walls made of six different materials, with and without lining of sound absorbing material.

To date, the sound insulation of acoustic enclosures for noise sources was determined with the use of parameters such as the sound power or pressure insulation in the frequency range below 10 kHz [18, 19]. The method for determining these quantities within the frequency range of 10–40 kHz is provided below. The rationale behind the need to work out such method was that in this frequency range, the value of the sound pressure insulation depends, to a large extent, on directional properties of the sound source. Therefore, the parameter that characterises the acoustic enclosure sound insulating properties more appropriately is the sound power insulation of enclosure (the use of the word “power” means that the sound insulation is calculated from the sound power level, the term being consistent with the commonly adopted standards EN ISO 11546-2:2009 and EN ISO 15667:2000 [18, 19]). The sound power insulation, on the analogy of [2, 3, 6–14, 16, 18, 19, 21, 28, 30], is determined in 1/3-octave frequency bands with centre frequencies *fi* (10, 12.5, 16, 20, 25, 31.5, and 40 kHz) according to the following formula:

1 $$ {D}_{W, fi}={L}_{W,\mathrm{with}\mathrm{out}, fi}-{L}_{W,\mathrm{with}, fi} $$

where:

*L*_*W*,without*,fi*_*, L*_*W*,with*,fi*_ (dB): the sound power level, in 1/3-octave frequency band with the centre frequency *fi*, for a laboratory sound source without acoustic enclosure and provided with the acoustic enclosure, respectively.

The source sound power level, as for the frequency below 10 kHz [22–24, 26] or below 20 kHz [25] (both inside and without the enclosure), is calculated using the following formula:

2 $$ {L}_{W, fi}=\left\langle {L}_{p, fi}^{\prime}\right\rangle -{K}_{\mathrm{bgn}, fi}-{K}_{\mathrm{env}, fi}+10{\log}_{10}\left(\left\{S\right\}\right)\mathrm{dB}+{K}_{\mathrm{air}, fi} $$

where:

〈*L′*_*p*,*fi*_〉 (dB): the average sound pressure level on the measurement surface encompassing the laboratory sound source (in 1/3-octave frequency band with the centre frequency *fi*),
*K*_bgn*,fi*_ (dB): the correction taking into account the background noise during the measurements (in 1/3-octave frequency band with the centre frequency *fi*),
*K*_env*,fi*_ (dB): the environmental correction in 1/3-octave frequency band with the centre frequency *fi*,
*K*_air*,fi*_ (dB): the air correction [9–12, 25, 29, 30] in 1/3-octave frequency band with the centre frequency *fi*,
*S* (m^2^): the measurement surface area.

**Fig. 1:**
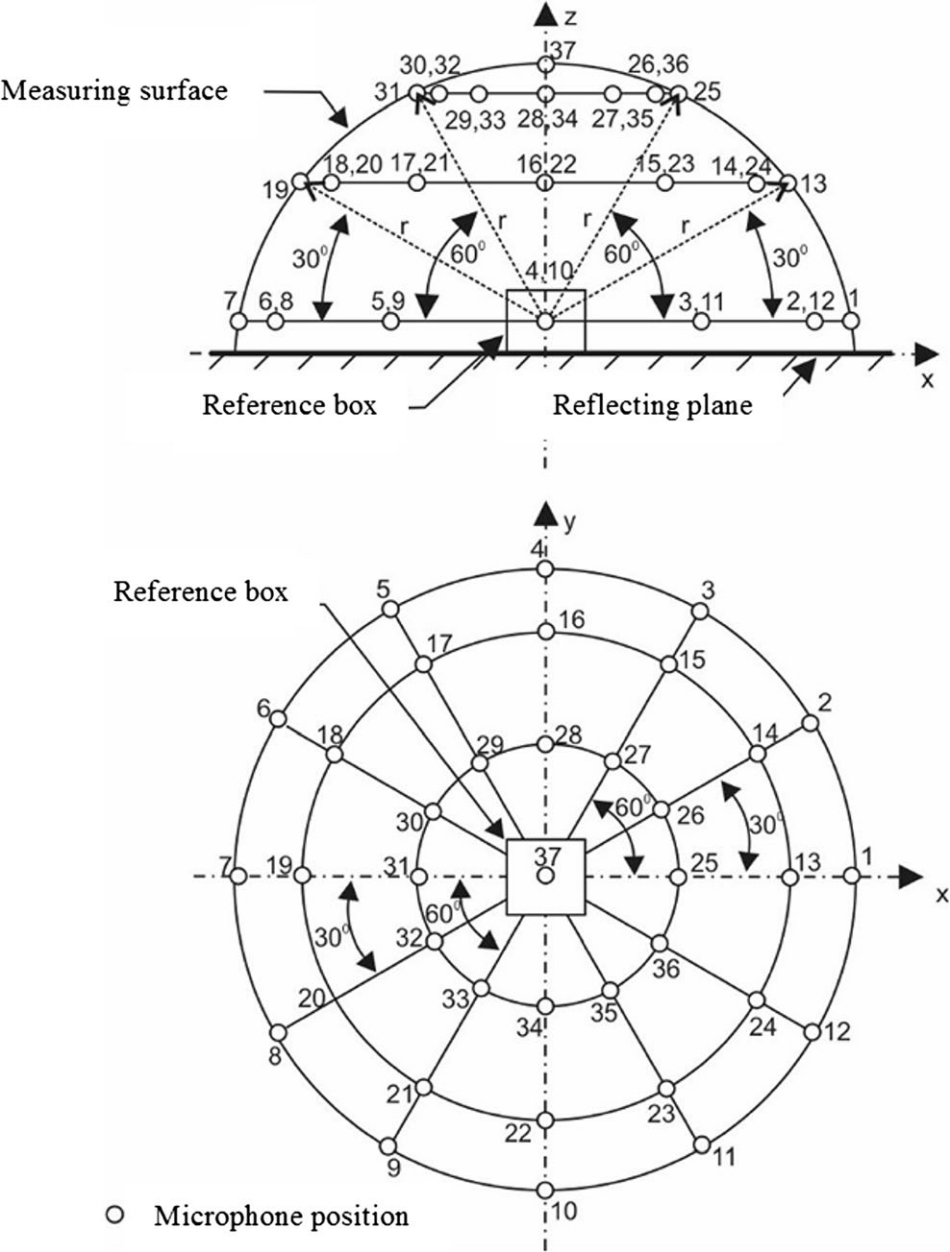
Distribution of 37 measurement points on a hemispherical measuring surface used for the determination of sound insulation properties of enclosures [9, 10, 30]

The average sound pressure level on the measurement surface is calculated in 1/3-octave frequency bands with centre frequencies *fi* (10, 12.5, 16, 20, 25, 31.5, and 40 kHz) according to the formula

3 $$ \left\langle L{\prime}_{p, fi}\right\rangle =10{\log}_{10}\left(\frac{1}{n}{\sum}_{j=1}^n{10}^{0.1\bullet {L}_{p, fi,j}}\right)\mathrm{dB} $$

where:

*n*: the number of measurement points,
*L*_*p,fi,j*_ (dB): the sound pressure level on the measurement surface at point number *j* (Fig. 1), in 1/3-octave frequency band with the centre frequency *fi*.

The correction taking account of the background noise *K*_bgn*,fi*_ is calculated in 1/3-octave frequency bands with centre frequencies *fi* (10, 12.5, 16, 20, 25, 31.5, and 40 kHz) with the use of the following formula:

4 $$ {K}_{\mathrm{bgn}, fi}=-10{\log}_{10}\left[1-{10}^{-0.1\left(\left\langle {L}_{p,\mathrm{src}, fi}\right\rangle -\left\langle {L}_{p,\mathrm{bgn}, fi}\right\rangle \right)}\right]\ \mathrm{dB} $$

where:

〈*L*_*p,*src*,fi*_〉 (dB): the average value of the sound pressure level, in 1/3-octave frequency band with centre frequency *fi*, on the measurement surface when the laboratory sound source is active, calculated from the formula of Eq. (3),
〈*L*_*p,*bgn*,fi*_〉 (dB): the average value of the sound pressure level, in 1/3-octave frequency band with centre frequency *fi*, on the measurement surface when the laboratory source is inactive, calculated from the formula of Eq. (3).

The background noise correction *K*_bgn*,fi*_ must not exceed 3 dB.

The environmental correction *K*_env*,fi*_ in rooms with a hemispherical measurement surface is calculated in 1/3-octave frequency bands with centre frequencies *fi* (10, 12.5, 16, 20, 25, 31.5, and 40 kHz) according to the formula

5 $$ {K}_{\mathrm{env}, fi}=10{\log}_{10}\left(1+4\frac{S}{A_{fi}}\right)\kern0.5em \mathrm{dB} $$

where:

*S* (m^2^): the measurement surface area (for a hemispherical measurement surface with radius *r, S =* 2*πr*^2^),
*A*_*fi*_ (m^2^): the sound absorption of the room, in 1/3-octave frequency band with centre frequency *fi*,
*V* (m^3^): the test room volume.

The environmental correction *K*_env*,fi*_ for the assumed hemispherical measurement surface in the room where tests were conducted is presented in Table 1.

**Table 1:** The environmental correction *K*_env*,fi*_ and the air correction *K*_air*,fi*_ for hemispherical measurement surface with radius *r* = 1 m in the room where measurements were taken [9–12, 25, 29, 30]

Frequency (kHz)	10	12.5	16	20	25	31.5	40
*K*_env*,fi*_ (for *r* = 1 m) (dB)	0.31	0.27	0.24	0.21	0.20	0.15	0.14
*K*_air*,*1m*,fi*_ (for humidity 50% and temperature 22 °C) (dB)	0.15	0.22	0.34	0.50	0.74	1.11	1.68

Calculations concerning the sound insulation of enclosures do not take into account the correction *K*_env*,fi*_ (it will be eliminated in the course of calculations). That does not mean that this value is of no importance. Small values of the environmental correction serve as an evidence that measurements were conducted in a quasi-free field, and thus in accordance with the basic assumption of the method. Therefore, the value of this specific correction should not exceed 4 dB (*K*_env*,fi*_ ≤ 4 dB, similarly as provided in EN ISO 9295 [25]).

The air correction *K*_air*,fi*_ at the distance of 1 m, i.e. *K*_air,1m*,fi*_, was estimated with the use of the extrapolation method on the basis of data from PN-EN ISO 9295 [25].

Because determination of the sound power insulation for an acoustic enclosure consists in calculation of differences between sound power levels on the same measurement surface in the same room, values of the air correction *K*_air*,fi*_ and the environmental correction *K*_env*,fi*_ do not affect the result of the sound insulation determination. Therefore, the sound insulation formula of Eq. (1) can be transformed into the following form:

6 $$ {\displaystyle \begin{array}{c}\begin{array}{c}\begin{array}{c}{D}_{W, fi}={L}_{W,\mathrm{with}\mathrm{out}, fi}-{L}_{W,\mathrm{with}, fi}=\\ {}=\left\langle {L}_{p,\mathrm{with}\mathrm{out}, fi}^{\prime}\right\rangle -{K}_{\mathrm{bgn},\mathrm{with}\mathrm{out}, fi}-{K}_{\mathrm{env}, fi}+10\log \left(\left\{S\right\}\right)\mathrm{dB}+{K}_{\mathrm{air}, fi}\end{array}\\ {}-\left(\left\langle {L}_{p,\mathrm{with}, fi}^{\prime}\right\rangle -{K}_{\mathrm{bgn},\mathrm{with}, fi}-{K}_{\mathrm{env}, fi}+10\log \left(\left\{S\right\}\right)\mathrm{dB}+{K}_{\mathrm{air}, fi}\right)\end{array}\\ {}=\kern0.5em \left\langle {L}_{p,\mathrm{with}\mathrm{out}, fi}^{\prime}\right\rangle -{K}_{\mathrm{bgn},\mathrm{with}\mathrm{out}, fi}-\left(\left\langle {L}_{p,\mathrm{with}, fi}^{\prime}\right\rangle -{K}_{\mathrm{bgn},\mathrm{with}, fi}\right)\\ {}=\left\langle {L}_{p,\mathrm{with}\mathrm{out}, fi}^{\prime}\right\rangle -\left\langle {L}_{p,\mathrm{with}, fi}^{\prime}\right\rangle +{K}_{\mathrm{bgn},\mathrm{with}, fi}\end{array}} $$

where:

*L*_*W,*without*,fi*_ and *L*_*W,*with*,fi*_ (dB): the sound power levels, in 1/3-octave frequency band with the centre frequency *fi*, for a laboratory sound source, without acoustic enclosure and provided with the enclosure, respectively,
*L′*_*p,*without*,fi*_ and *L′*_*p,*with*,fi*_ (dB): the average sound pressure levels on the surface encompassing the laboratory sound source, in 1/3-octave frequency band with the centre frequency *fi*, without and with acoustic enclosure, respectively, calculated from the formula of Eq. (3),
*K*_bgn*,*without_*,*_*fi*_ and *K*_bgn*,*with*,fi*_ (dB): corrections for the background noise during the measurements, in 1/3-octave frequency band with the centre frequency *fi*, for the laboratory source without and with acoustic enclosure, respectively, calculated from the formula of Eq. (4) (in any case, *K*_bgn*,*with*,fi*_ must not exceed 3 dB, and since the difference of the average sound pressure level on the measurement surface during measurements concerning the source without acoustic enclosure in the tested room and the measurements of the background noise in the considered frequency bands exceeded 15 dB, *K*_bgn*,*without*,fi*_ can be omitted because it does not affect the final result),
*K*_env*,fi*_ (dB): the environmental correction in 1/3-octave frequency band with the centre frequency *fi* (can be omitted because it does not affect the result but in any case, *K*_env*,fi*_ must not exceed 4 dB),
*S* (m^2^): the measurement surface area (which does not affect the final result).

The tests were conducted in a room with high sound absorption (semi-anechoic room) and a highly reflective floor (Fig. 1). The tests were conducted above the reflective surface.

The number of measurement points at which the sound pressure level on the measurement surface was measured was assumed to be 37 (in accordance with the requirements of EN ISO 3744 [24], whereas according to EN ISO 9295 [25], for the technical method, the number of measurement points must be greater than the maximum difference of sound pressure level values on the measurement surface. From the present author’s own research concerning determination of the minimum number of measurement points for the purpose of evaluation of the sound power level for ultrasonic noise sources [9, 10], the minimum number of measurement points is 20. In cases when the condition established in EN ISO 3744 [24] is not met, the number of measurement points should be increased. The set of 37 measurement points can be distributed on the hemispherical measurement surface every 30° of the plane angle, both in the horizontal and vertical plane (Fig. 1).

**Fig. 2:**
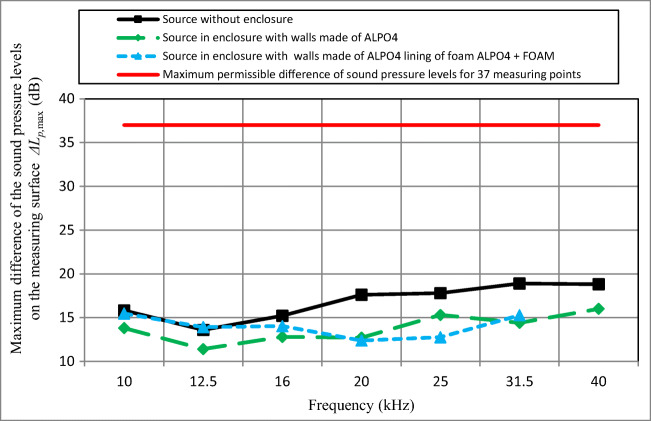
The maximum difference in the sound pressure levels on the measuring surface *ΔL*_*p*,max_ for the laboratory source without acoustic enclosure, with acoustic enclosure with walls made of ALPO4, and with enclosure with walls made of ALPO4 lining of foam ALPO4 + FOAM

In Fig. 2, results of measurement of maximum differences in the sound pressure level on the measuring surface *ΔL*_*p*,max_ are presented. For the entire frequency range, the maximum difference in the sound pressure levels on the measuring surface *ΔL*_*p*,max_ did not exceed 20 dB and was significantly lower than the allowable value of 37 dB. It stems from this that the number of 37 measurement points adopted for the tests is representative and has been selected in a correct manner.

**Fig. 3:**
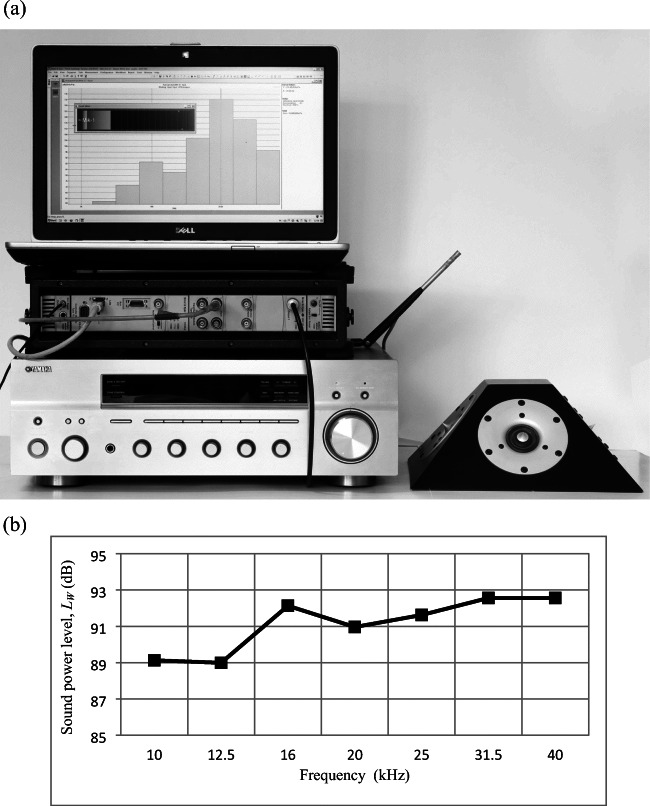
Laboratory equipment used for measurements in the frequency range 10–40 kHz: **a** equipment (described above in the text), **b** the measured sound power level

The laboratory equipment used for the tests carried out in the frequency range of 10–40 kHz included (Fig. 3):

- in the sound generating portion: noise generator 8–50 kHz PULSE type 3560C with PULSE multi-analyser (Brüel & Kjær), RX 797 power amplifier (Yamaha), and self-made column loudspeaker in the form of a truncated square-based pyramid with 4 R2904–7000 loudspeakers (ScanSpeak);
- in the sound-measuring portion: 1/4-in. microphone with type 4944A preamplifier, input/output module 3110 type 3560C, and Data-Software system for PULSE Types LabShop type 7700 (all from Brüel & Kjær).

## Test results

### The sound power insulation of skeleton acoustic enclosures with walls made of different materials (without sound-absorbing materials inside)

**Fig. 4:**
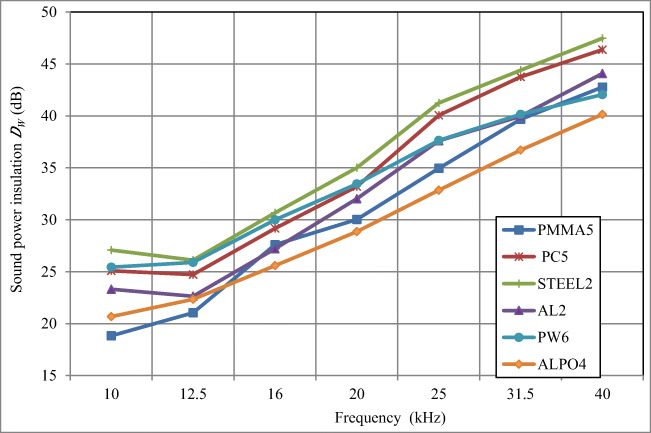
The sound power insulation *D*_*W*_ of skeleton acoustic enclosures with walls made of different materials and without any sound-absorbing materials inside (results for PW6 were published in [30])

In Fig. 4 and in Table 2, results of determination of the sound power insulation are given for skeleton enclosures (without any lining on their inner surface) with walls made of PMMA5, PC5, STEEL2, AL2, PW6, and ALPO4.

**Table 2:** The effect of increasing sound frequency on the sound power insulation *ΔD*_*W*_ of skeleton acoustic enclosures with walls made of different materials

Material used for enclosure walls	Average increase of sound power insulation *ΔD*_*W*_ for the frequency increase (dB)	
	by an octave	by 1/3 of an octave
PMMA5 — single-layer PMMA poly(methyl methacrylate), wall thickness 5 mm	12.5	4.0
PC5 — single-layer PC polycarbonate, wall thickness 5 mm	12.8	3.5
STEEL2 — single-layer sheet steel, wall thickness 2 mm	12.3	3.4
AL2 — single-layer sheet aluminium, wall thickness 2 mm,	12.1	3.5
PW6 — single-layer plywood, wall thickness 6 mm	9.6	2.8
ALPO4 — three-layer composite (sheet aluminium 0.3 mm + polyethylene 3.4 mm + sheet aluminium 0.3 mm)	10.3	3.2

The values of the sound power insulation of skeleton enclosures with walls made of these different materials were included in the range from 18.8–27.1 dB (for the frequency of 10 kHz) to 40.2–47.5 dB (for frequency of 40 kHz), as shown in Fig. 4.

In the frequency range under consideration, a monotonic increase of the sound power insulation *D*_*W*_ value with increasing sound frequency was observed. For enclosure with walls made of PMMA5, PC5, STEEL2, and AL2, the sound power insulation *D*_*W*_ increased by about 12.5 dB, and for walls made of PW6 and ALPO4 by about 10 dB for doubled sound frequency, i.e. per an octave (Table 2). The average increase of the sound power insulation *D*_*W*_ corresponding to the sound frequency increased by 2^1/3^ (per 1/3 of an octave) for acoustic enclosure walls made of PMMA5, PC5, STEEL2, and AL2 was about 3.5 dB, compared to about 3 dB for enclosures with walls made of PW6 and ALPO4.

The highest sound power insulation *D*_*W*_ was observed for the acoustic enclosure with steel walls of 2 mm thick STEEL2 (27.1–47.5 dB in the frequency range of 10–40 kHz, Fig. 4). The lowest sound power insulation *D*_*W*_ was measured for the acoustic enclosure with walls made of ALPO4 composite (sheet aluminium 0.3 mm + polyethylene 3.4 mm + sheet aluminium 0.3 mm) and amounted to 20.7–40.2 dB in the frequency range of 10–40 kHz. In the frequency bands centred at of 10 and 12.5 kHz, a slightly lower sound power insulation *D*_*W*_ was observed for the acoustic enclosure with walls made of PMMA5, where the value of 18.8 dB was measured for the frequency of 10 kHz.

The maximum differences in the sound power insulation *D*_*W*_ for the tested enclosure were included within the bracket of 5.1–8.4 dB (for each frequency within the range of 10–40 kHz). This means that for an ultrasonic sound source emitting the sound energy in a narrow frequency band (tonally), the sound power insulation of an acoustic skeleton enclosure may vary by from about 5 to about 8 dB depending on the material used for the walls. Therefore, the sound pressure level in the vicinity of enclosures with walls of different materials will be by 5 to 8 dB higher or lower, depending on the materials used for the enclosure walls. The difference, not large as it may seem, can be nevertheless considered important because it can result in a reduction or an increase of the human exposure to noise by 5–8 dB.

The above conclusions concern enclosures without any sound-absorbing material used inside, in particular, on enclosure internal walls.

### The sound power insulation of skeleton acoustic enclosures with walls made of aluminium and plywood of various thickness (without sound-absorbing materials inside)

**Fig. 5:**
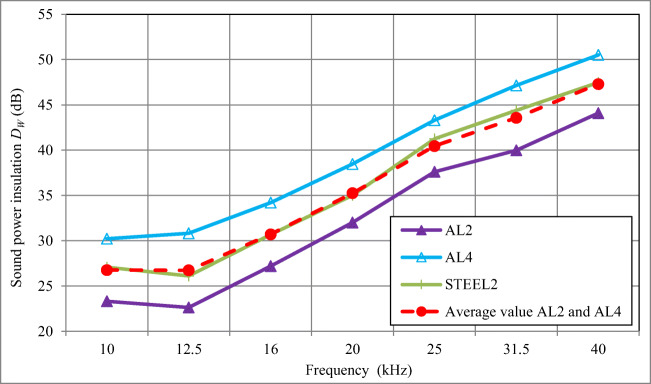
The sound power insulation *D*_*W*_ for skeleton enclosures with walls made of single-layer 2 mm and 4 mm sheet aluminium, with average values compared to those obtained for enclosure of single-layer 2 mm sheet steel

In Fig. 5, the sound power insulation *D*_*W*_ of skeleton acoustic enclosures made of sheet aluminium with 2 mm and 4 mm thick walls (AL2 and AL4) is plotted. For the sake of comparison, results of the same test with the skeleton acoustic enclosure made of steel with 2 mm thick walls (STEEL2) are also provided.

The sound power insulation of the skeleton acoustic enclosure made of 2 mm aluminium AL2 is lower than this measured for the acoustic enclosure made of steel STEEL2 by about 3.6 dB (3.0–4.4 dB in the frequency range under consideration; Fig. 5). The arithmetic average value of the sound power insulation *D*_*W*_ value for the enclosure made of 2 mm AL2 and 4 mm AL4 aluminium (the so-called predicted value for the acoustic enclosure with 3 mm thick walls) is almost identical to the sound power insulation *D*_*W*_ of the enclosure made of 2 mm steel STEEL2. This proves that in the frequency range of 10–40 kHz, to obtain similar values of the sound power insulation *D*_*W*_ for the enclosure made of steel and aluminium, the walls of the latter should be twice as thick which, however, would still make the aluminium enclosures about half as light as the steel ones (the density of aluminium is 2720 kg/m^3^ compared to 7500–7900 kg/m^3^ for various steel grades).

**Fig. 6:**
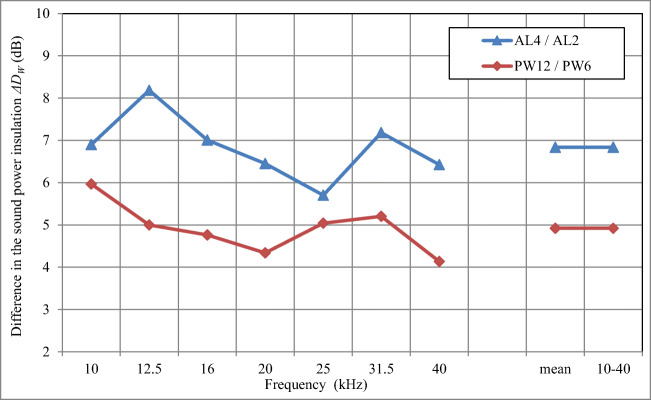
The difference in the sound power insulation *ΔD*_*W*_ for walls of skeleton enclosures made of 4 mm and 2 mm thick aluminium and for walls made of 12 mm and 6 mm thick plywood (results of sound power insulation for the skeleton enclosure made of PW6 and PW12 were published in [30])

In the frequency range of 10–40 kHz, the sound power insulation *D*_*W*_ of the skeleton acoustic enclosure made of sheet aluminium with 4 mm thick walls (AL4) is higher than the sound insulation of the skeleton acoustic enclosure made of sheet aluminium with 2 mm thick walls (AL2) by 6.8 dB on average (5.7–8.2 dB; Fig. 6). From this it follows that for doubled thickness of aluminium walls (and the corresponding two-fold increase of the enclosure mass), the increase of the sound power insulation *D*_*W*_ by about 7 dB is observed (Fig. 6). Analogously, two-fold increase of plywood walls thickness (doubled acoustic enclosure mass) results in the increase of the sound power insulation *D*_*W*_ by about 4.9 dB (Fig. 6). Most probably, the larger impact of the increase of aluminium walls thickness compared to the increase of plywood walls thickness on the acoustic enclosure sound power insulation *D*_*W*_ is caused by, apart from the mass (the density of aluminium and wood is 2720 kg/m^3^ and about 600 kg/m^3^, respectively), the higher rigidity of thicker aluminium walls (4 mm).

The conclusions from the above analysis apply also to acoustic enclosures without the sound-absorbing material used inside.

### The sound power insulation of skeleton acoustic enclosures with walls made of different materials with sound-absorbing materials on the inside walls

**Fig. 7:**
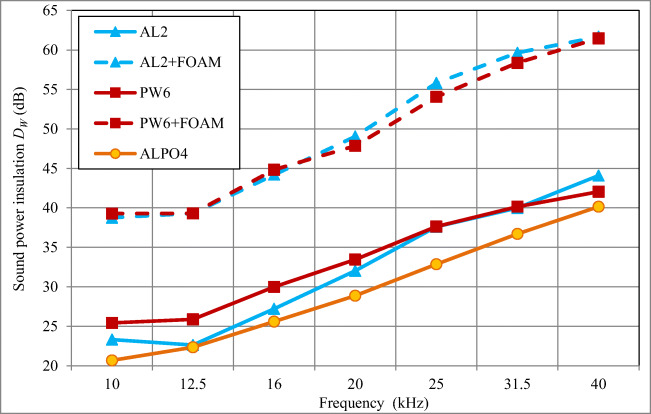
The sound power insulation *D*_*W*_ of skeleton acoustic enclosures with walls made of aluminium AL2, plywood PW6, and composite ALPO4 (sheet aluminium 0.3 mm + polyethylene 3.4 mm + sheet aluminium 0.3 mm) with and without 15 mm foam

**Fig. 8:**
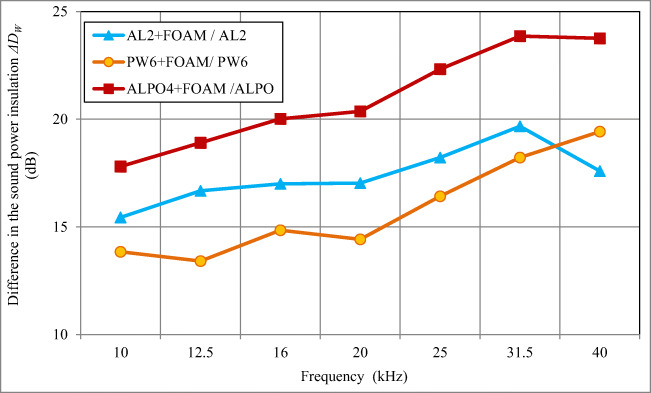
The difference in sound power insulation *ΔD*_*W*_ for the walls of the skeleton enclosure with and without 15 mm foam, made of aluminium AL2, plywood PW6 and composite ALPO4 (aluminium 0.3 mm + polyethylene 3.4 mm + aluminium 0.3 mm)

In Figs. 7 and 8, results of determination of the sound power insulation *D*_*W*_ are given for skeleton acoustic enclosures with 2 mm thick aluminium walls (AL2), 6 mm thick plywood walls (PW6), and 4 mm thick composite walls (ALPO4) lined inside with 15 mm thick polyurethane foam.

The maximum difference in the sound power insulation *D*_*W*_ for skeleton enclosures with walls made of 2 mm aluminium (AL2), 6 mm plywood (PW6), and 4 mm composite (ALPO4), all of which were lined with a sound-absorbing material (15 mm polyurethane foam) inside, depending on the frequency (in the range of 10–40 kHz), remained within the range of 0.79–2.44 dB (1.7 dB on average; Fig. 7). Taking into consideration that the sound power insulation *D*_*W*_ of such enclosures (with sound-absorbing foam inside) was included within the range of 38.5–64 dB, it can be stated that the use of inner foam lining masked the effect of various sound insulation properties of acoustic enclosures (without any sound-absorbing material inside) resulting from different materials they were made of.

The average increase of the sound insulation of acoustic enclosures in the frequency range of 10–40 kHz after application of a 15 mm foam lining on their inside walls (Fig. 8) was as follows: 17.4 dB (15.4–19.7 dB) for 2 mm thick aluminium walls (AL2); 15.8 dB (13.4–19.4 dB) for 6 mm thick plywood walls (PW6); and 21.0 dB (17.8–23.9 dB) for 4 mm thick composite walls (ALPO4).

To recapitulate, the average sound power insulation *D*_*W*_ of the skeleton acoustic enclosure made of sheet aluminium with 2 mm thick walls (AL2) in the frequency range of 10–40 kHz was 32.4 dB (with 15 mm foam, it increased by an additional 17.4 dB); for plywood enclosure with 6 mm thick walls (PW6) it was 33.5 dB (with 15 mm foam, it increased further by 15.8 dB); and for composite enclosure with 4 mm thick walls (ALPO4) it was 29.6 dB (with 15 mm foam lining, it increased by another 21.0 dB).

## Conclusion

Enclosures for noise sources emitting the sound energy in the frequency range of 10–40 kHz constitute an effective measure of noise reduction. Leak-proof (in other words, fully closed or sealed) enclosures, without the sound-absorbing material inside, reduce the sound pressure level in the environment (expressed as the sound insulation of acoustic enclosures) by from about 19–27 dB (for the frequency of 10 kHz) to about 40–48 dB (for the frequency of 40 kHz).

Among the tested enclosures without the sound-absorbing material inside (with 2 mm thick sheet aluminum walls, 2 mm thick sheet steel walls, 5 mm thick plexiglass walls, 5 mm thick polycarbonate walls, and 4 mm thick aluminum-polyethylene composite walls), the highest sound insulation of acoustic enclosures, ranging from 27 dB to 48 dB for the frequency of 10 kHz and 40 kHz, respectively, was observed for the steel acoustic enclosure, whereas the lowest insulation values (21–40 dB) were obtained for the composite acoustic enclosure.

The sound insulation of acoustic enclosures increased with increasing frequency by 10–12 dB per octave and with increasing wall thickness by about 5–7 dB per doubled mass.

Applying a sound-absorbing material covering acoustic enclosure walls on the inside results in a significant increase in the sound insulation. It can be estimated that in the tested cases, the resultant average, within the frequency range of 10–40 kHz, of the value of sound insulation of the skeleton enclosure made of 2 mm thick sheet aluminium was 32.4 dB without and 49.8 dB with the sound-absorbing material inside; for the acoustic enclosure made of plywood with 6 mm thick walls — 33.5 and 49.3 dB, respectively; and for the acoustic enclosure made of 4 mm thick composite — 29.6 dB and 50.6 dB, respectively. To generalise the above results it can be stated that the use of sound-absorbing material inside an acoustic enclosure results in a 32–41% increase in the sound power insulation of the enclosure. This evidences the vital importance of using sound-absorbing materials inside enclosures for ultrasonic noise sources (emitting sound in the frequency range of 10–40 kHz).

The described research results allow to select materials from which sound-insulating enclosures for ultrasonic noise emitting devices are to be made. The results can be also used to estimate the ultrasonic noise level reduction achieved at workstations (and thus to predict the risks related to exposure of workers to ultrasonic noise). In case of using sound-insulating enclosures with different dimensions and/or structure and/or made out of materials different from those subjected to testing, it would be necessary to measure the sound insulation of such enclosures. The present study, in the opinion of the author, has an application potential but at the same time, it can be considered an introduction to a more detailed analysis of the possibility to reduce the hazards related to the ultrasonic noise at workstations. In that area, its seems to be appropriate to undertake the future research effort in three specific areas. The first of such research topics would concern ultrasonic devices and include: developing a method for determination of ultrasonic source noise emissions (the sound power level); determining the noise emission for typical ultrasonic noise sources; and undertaking legislative activities in the scope of ultrasonic noise emission of sources (and possibly restrictions for noise emission from such sources, on the analogy of legal instruments applicable to audible noise i.e. in directives 2006/42/EC and 2005/88/EC [22, 23] and international standards EN ISO 3744:2010 and EN ISO 9295:2015 [24, 25]). Another field of research would pertain to sound-absorbing materials and include: establishing a method for determining sound-absorbing properties of materials in the ultrasonic noise frequency range (the sound absorption coefficient); determining properties of sound-absorbing materials used for inner linings of sound insulating enclosures in the ultrasonic noise frequency range; and initiating legislative activities in the scope of sound-absorbing properties of materials in the frequency range 10–40 kHz (on the analogy of regulations currently applicable to the frequency range 100–5000 Hz). The third area of research would concern soundproof barriers and enclosures (for ultrasonic noise sources), and in particular: developing methods for determining insulation properties of sound-insulating enclosures and barriers used for enclosure walls in the ultrasonic noise frequency range (the sound insulation); determining acoustic insulation properties of sound-insulating enclosures and barriers in the ultrasonic noise frequency range; and entering into legislative activities in scope of manufacturers’ declarations concerning sound insulation properties of enclosures and barriers in the ultrasonic noise frequency range (on the analogy of EN ISO 11546-2:2009 and EN ISO 15667:2000 [18, 19] currently applicable to lower frequencies).

## Data Availability

Not applicable.
